# Epidemiology of primary brain tumor among adolescents and adults in Palestine: a retrospective study from 2018 to 2023

**DOI:** 10.1186/s12883-024-03677-1

**Published:** 2024-05-23

**Authors:** Mohammad Abuawad, Ahmed Daqour, Abdulsalam Alkaiyat, Ahmad Rjoub, Wafaa Abu Zahra, Noor Issa, Yazan Dumaidi, Shahed Nasser

**Affiliations:** 1 Department of Biomedical Sciences, Faculty of Medicine and Health Sciences, An-Najah National University, Nablus, Palestine; 2 Almakassed Hospital, Al-Quds University, Jerusalem, Palestine; 3 Department of Medicine, Faculty of Medicine and Health Sciences, An-Najah National University, Nablus, Palestine; 4 Rafeedia Surgical Hospital, Nablus, Palestine; 5 Faculty of Medicin, University of Jordan, Amman, Jordan

**Keywords:** Brain, Tumors, Palestine, Incidence, Histopathology

## Abstract

**Backgrounds:**

Primary brain tumors (PBTs) are uncommon, but they significantly increase the risk of disability and death. There is a deficiency of data concerning the epidemiology and anatomical distribution of PBTs among adults in Palestine.

**Methods:**

A retrospective descriptive study in which data were collected from the clinical reports of Palestinian patients diagnosed with PBTs at Al-Makassed Hospital during the period (2018–2023).

**Results:**

In Palestinian adolescents and adults, the incidence rate of PBTs was 3.92 per 100,000 person-years. Glioblastoma (18.8%) was the most common type identified, and it was more common in males. Non-malignant tumors were more common than malignant tumors (2.41 vs. 1.52 per 100,000). The mortality rate from PBTs was 4.8%. The most common initial symptom was headaches, and it occurred more with non-malignant tumors (57.28% vs. 42.72%, *p*-value < 0.001). Cerebral meninges (26.3%) were the most common location for primary brain tumors (*p*-value < 0.001).

**Conclusion:**

This is the first study of primary brain tumor epidemiology in Palestine. The overall incidence of PBTs in Palestinian adolescents and adults was 3.96 per 100,000, which was lower than the incidence rate of primary brain tumors worldwide. More studies on the epidemiology and distribution of PBTs in Palestine are recommended.

## Background

A total of more than 150 different types of brain tumors have been reported in the literature; the definitions of “primary” and “metastatic” refer to the two main groups [1, 2]. While metastatic brain tumors originate in areas other than the brain (such as the breast or lungs) and then spread to the brain, usually through the bloodstream, primary brain tumors (PBTs) originate from the brain or its immediate environs. PBTs can be either benign or malignant, despite having different origins. The most prevalent malignant PBT is glioblastoma with incidence of 3.21 per 100,000 person in the United States [3]. It is perceived that damage to particular genes on a cell’s chromosomes causes abnormal functioning, which in turn leads to the development of brain tumors. Usually, these genes’ self-destruction systems help fix gene errors and regulate the rate at which cells divide [4].

PBTs are a broad category that includes a range of benign and malignant tumors that arise from different areas of the brain, including the skull base, meninges, cranial nerves, and the parenchyma [5, 6]. Over the decades, multiple studies have been implemented to determine the major risk factors contributing to the etiology of PBT; nevertheless, it is unclear which main risk factor accounts for a significant fraction of instances in the etiology of the brain and other central nervous system tumors [7].

PBTs are relatively rare, but they significantly increase the risk of disability and death. As a consequence, patients, their families, and the healthcare system bear a heavy burden [5]. About 33.4% of PBTs survive for five years [8]. In Palestine, in 2021, these tumors have contributed to roughly 5.7% of all cancer-related deaths [9].

Brain and other central nervous system (CNS) tumors constitute the 17th most prevalent type of cancer worldwide, contributing to 1.7% of all cancer cases (excluding non-melanoma skin cancer) [10]. In 2015, PBTs comprised 7% and 6% of all cancer forms that affected males and females in Palestine respectively. In contrast, the rate of brain cancer as of 2020 was 3.1% for both sexes [9]. Nevertheless, no such study provided reliable data on the types of these brain tumors, their epidemiology, clinical, anatomical, and histological presentations, and outcomes.

The epidemiologic features and anatomical distribution of adult PBTs are not well understood in Palestine. In this study, we aim to examine the incidence rate of adolescents and adult PBTs in Palestine in the last six years (2018–2023) and to determine their epidemiologic characteristics, histologic types, and anatomical distribution.

## Methods

### Study design and setting

Our study was a retrospective epidemiological descriptive study in which we collected data from the histopathology reports and MRI reports of Palestinian patients diagnosed with PBTs who were treated at Al-Makassed Hospital in Jerusalem, the largest tertiary referral center for PBTs in Palestine during the period (2018–2023). Our targeted population is patients diagnosed with PBTs who received healthcare at Al-Makassed Hospital in Jerusalem during this period.

### Inclusion and exclusion criteria

Palestinian patients with PBTs aged 15 or more, whether diagnosed at Al-Makassed Hospital or referred to this hospital for treatment included in this study. Patients below the age of 15 years old, or with metastatic (secondary) brain tumors, spinal cord tumors, and those with missing histopathology reports or MRI reports were excluded.

### Variables

The variables included in this study were the age at the time of diagnosis, sex (male or female), type of residency (city, village, or camp), anatomical location of the tumor in the brain as stated in the neuroradiology reports, type of the tumor on histopathology, and the behavior of the tumor (either benign, borderline, or malignant). The histopathological type of the tumor and its anatomical location within the brain were coded based on the third edition of the International Classification of Diseases of Oncology (ICD-O-3) Manual [11]. The histopathological type and behavior of the tumor were coded in accordance with the 2016 WHO Classification for CNS tumors [12].

### Data collection and tool

The main source of data collection was the clinical reports of patients for the sociodemographic data, the histopathology reports for the pathological type of the tumor, and the neuroimaging reports for the anatomical location of the tumor. The clinical reports and histopathology reports were obtained from the registry system at Al-Makassed Hospital. The population sizes for each year were obtained from the Palestinian Central Bureau of Statistics. The population size was used to calculate the crude incidence rate for each year.

### Data analysis

The 26.0 version of the Statistical Package for Social Sciences (SPSS) was used in the analysis. For categorical and continuous variables, a basic descriptive analysis was performed. The incidence was calculated per 100,000. Chi-square tests and cross-tabulation were used for the analysis of the categorical variables. Statistical significance was set at 5%, and any p-value below 0.05 was considered statistically significant.

## Results

### Sociodemographic characteristics of the patients

A total of 764 patients were diagnosed with PBTs during the period from 2018 to 2023. The patients exhibited a mean age of 44.58 years (SD ± 15.27). Females accounted for 53% of the total patients, whereas males constituted the remaining 47%. Geographically, about 49% of the patients were living in the major Palestinian cities, 44.1% in villages, and 6.9% in refugee camps. Regionally, 56.2% of the patients were from the West Bank and 43.8% from the Gaza Strip. The sociodemographic characteristics of the patients are presented in Table 1.

**Table 1:** Sociodemographic characteristics of the patients

Variable	Frequency (*n*)/Mean	Percentage (%)/SD
Age at diagnosis	44.58	± 15.27
*Sex*		
Male	359	47%
Female	405	53%
*Type of residency*		
City	374	49%
Village	337	44.1%
Refugee camps	53	6.9%
*Place of residency*		
West bank	429	56.2%
Gaza strip	335	43.8%
Total	764	100%

### Incidence rate for PBTs by years and sex

**Fig. 1:**
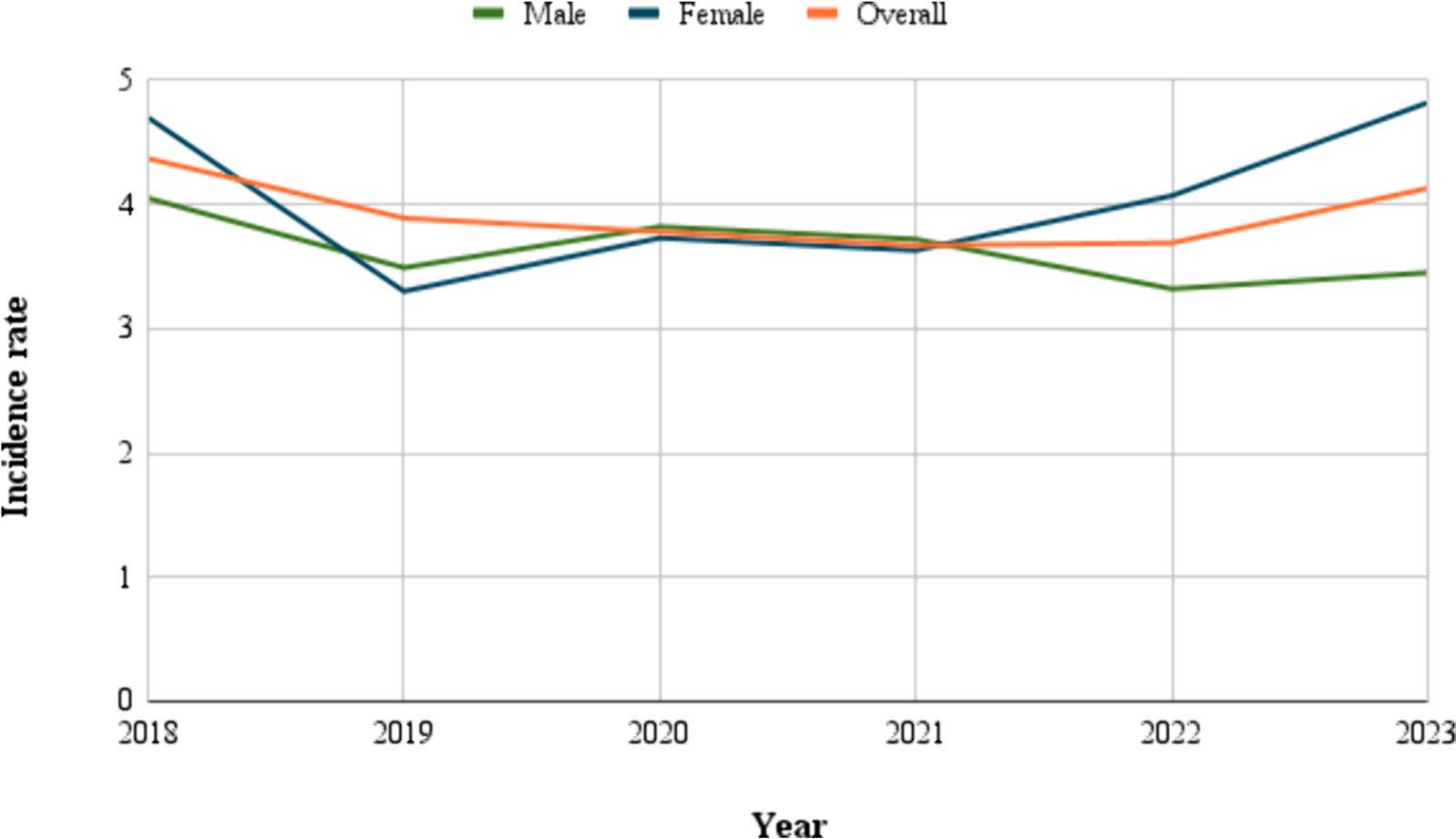
Annual Incidence rates of primary brain tumors per 100,000, classified by sex, for the period spanning from 2018 to 2023

During the period from 2018 to 2023, the annual incidence rate for PBTs ranged from 3.67 to 4.13, with an average annual incidence rate of 3.92. The overall incidence rate for 2018 was the highest, then abruptly declined to reach 3.89 in 2019, continued to gradually decrease until 2022, then increased to 4.13 in 2023. The average annual incidence rate for females was 4.21, higher than the average overall annual incidence rate (3.92) and the average annual incidence rate for males (3.64). The average annual male-to-female ratio was 0.86. The crude annual incidence rates for the PBTs are presented in Table 2 and illustrated in Fig. 1.

**Table 2:** Incidence rate by years and sex

Year	Number of cases	Population size	Crude Incidence rate^a^	Male population size	Incidence rate in males^a^	Female population size	Incidence rate in females^a^	M: F ratio
2018	132	3,020,527	4.37	1,529,715	4.05	1,490,812	4.70	1:1.16
2019	121	3,108,852	3.89	1,574,481	3.49	1,534,371	3.30	1.06:1
2020	121	3,200,943	3.78	1,621,087	3.82	1,580,306	3.73	1.02:1
2021	121	3,293,035	3.67	1,667,694	3.72	1,625,341	3.63	1.02:1
2022	125	3,388,842	3.69	1,716,144	3.32	1,672,698	4.07	1:1.23
2023	144	3,486,936	4.13	1,765,724	3.45	1,721,212	4.82	1:1.4
**Average**	-	-	3.92	-	3.64	-	4.21	1:1.16

^a^per 100,000

### Distribution of cases in age groups and sex groups

**Fig. 2:**
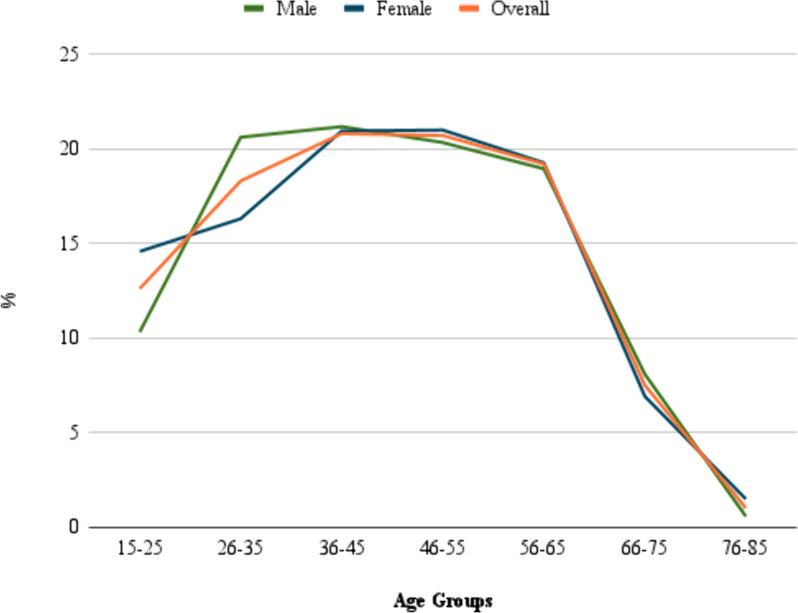
Distribution of primary brain tumor cases by age and sex

About 20.8% of the patients were aged from 36 to 45 years, and 20.7% of them aged from 46 to 55 years. Patients aged from 15 to 25 years were about 12.6% of the total patients included in the study. The percentages of patients increase gradually from the 15–25 age group to the 36–45 age group, then gradually decrease to the 66–75 age group. Only 1% of the patients aged between 76 and 85 years. No significant differences between males and females in the different age groups were found. The distribution of cases in age groups is shown in Table 3 and illustrated in Fig. 2.

**Table 3:** Distribution of cases in age groups and sex groups

Age groups	Frequency (*n*)	Percentage (%)	Male	Female	*p*-value
15–25	96	12.6	37 (10.31%)	59 (14.57%)	0.352
26–35	140	18.3	74 (20.61%)	66 (16.30%)	
36–45	159	20.8	76 (21.17%)	83 (20.94%)	
46–55	158	20.7	73 (20.33%)	85 (20.99%)	
56–65	146	19.2	68 (18.94%)	78 (19.26%)	
66–75	57	7.5	29 (8.08%)	28 (6.91%)	
76–85	8	1	2 (0.56%)	6 (1.48%)	

### The behavior of the PBTs based on the WHO classification

About 48.56% of the total PBTs were benign, 38.61% of the tumors were malignant, and 12.83% of the tumors were unspecified, borderline, or uncertain behavior. The incidence rate of malignant tumors was 1.52 and the incidence rate of non-malignant tumors was 2.41. The percentage of malignant tumors was higher in males compared to females (*p*-value < 0.001), and in patients living in villages compared to patients living in cities and refugee camps (*p*-value = 0.037). Benign tumors were more likely to occur in female patients compared to male patients (*p*-value < 0.001), and patients living in villages compared to patients living in cities and refugee camps (*p*-value = 0.037). The behavior of the PBTs is shown in Table 4.

**Table 4:** The behavior of the primary brain tumors based on the WHO classification

Variable	Benign	Unspecified, borderline, or uncertain behavior	Malignant	*p*-value
Sex:				
Male	142 (39.55%)	45 (12.53%)	172 (47.91%)	< 0.001
Female	229 (56.54%)	53 (13.09%)	123 (30.37%)	
**Type of residency**:				
City	161 (43.05%)	53 (14.17%)	160 (42.87%)	0.037
Village	158 (50.97%)	39 (12.58%)	113 (63.45%)	
Refugee Camp	25 (47.17%)	6 (11.32%)	22 (41.51%)	
Total	371 (48.56%)	98 (12.83%)	295 (38.61%)	

### Anatomical location

**Fig. 3:**
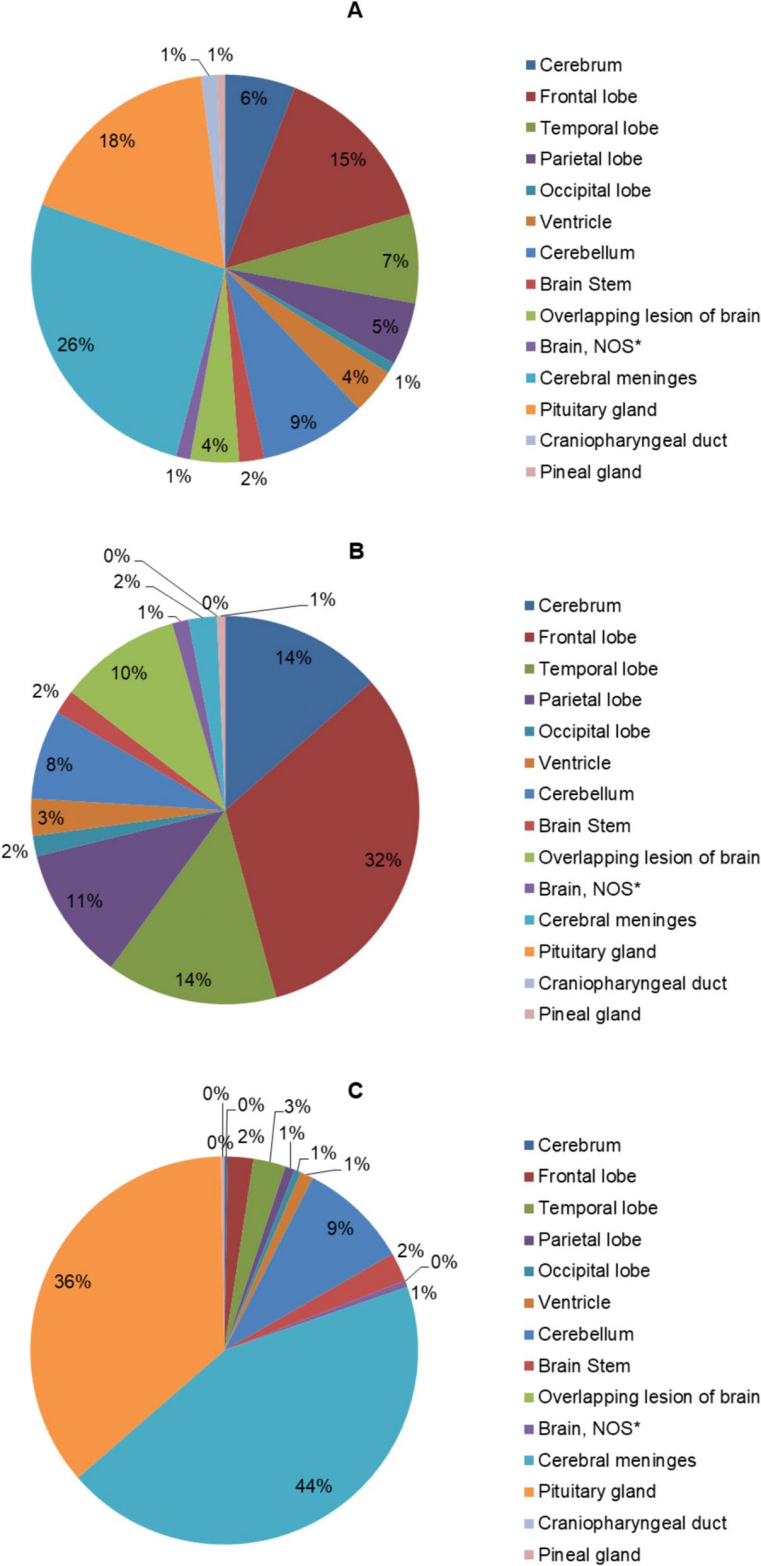
Distribution of primary brain tumors based on the anatomical location. Distribution of all primary brain tumors (**A**), distribution of malignant primary brain tumors (**B**), and distribution of benign primary brain tumors (**C**)

The most common locations for PBTs were the cerebral meninges (26.3%), pituitary gland (17.7%), and frontal lobe (14.5%). While the least common site for PBTs was the pineal gland (0.7%). About 37.8% of the PBTs were located in the cerebrum, cerebral cortex, and ventricles. The most common locations for the malignant PBTs were the frontal lobe (32.2%), temporal lobe (14.2%), and cerebrum (13.6%). Moreover, the most common locations for benign PBTs were the cerebral meninges (43.9%), pituitary gland (36.1%), and cerebellum (9.2%). The anatomical locations of the PBTs are presented in Table 5 and illustrated in Fig. 3.

**Table 5:** Distribution of the primary brain tumors by the anatomical location

Site	ICD-O-3 Site Code	*n*	(%)	Benign	Unspecified, borderline, or uncertain Behavior	Malignant	*p*-value
Cerebrum	C71.0	45	5.9	1 (2.22%)	4 (8.89%)	40 (88.89%)	<0.001
Frontal lobe	C71.1	111	14.5	8 (7.21%)	8 (7.21%)	95 (85.59%)	
Temporal lobe	C71.2	57	7.5	10 (17.54%)	5 (8.77%)	42 (73.68%)	
Parietal lobe	C71.3	40	5.2	3 (7.5%)	4 (10%)	33 (82.5%)	
Occipital lobe	C71.4	7	0.9	2 (28.57%)	0 (0%)	5 (71.43%)	
Ventricle	C71.5	29	3.8	4 (13.79%)	16 (55.17%)	9 (31.03%)	
Cerebellum	C71.6	68	8.9	34 (50%)	12 (17.65%)	22 (32.35%)	
Brain Stem	C71.7	16	2.1	9 (56.25%)	1 (6.25%)	6 (37.5%)	
Other brain							
Overlapping lesion of brain	C71.8	31	4.1	0 (0%)	1 (3.23%)	30 (96.77%)	
Brain, NOS^a^	C71.9	9	1.2	2 (22.22%)	3 (33.33%)	4 (44.44%)	
Cerebral meninges	C70.0	201	26.3	163 (81.09%)	31 (15.42%)	7 (3.48%)	
Pituitary and craniopharyngeal duct							
Pituitary gland	C75.1	135	17.7	134 (99.26%)	1 (0.74%)	0 (0%)	
Craniopharyngeal duct	C75.2	10	1.3	0 (0%)	10 (100%)	0 (0%)	
Pineal gland	C75.3	5	0.7	1 (20%)	2 (40%)	2 (40%)	

^a^*NOS* Not Otherwise Specified

### Distribution of the PBTs by histopathology

**Fig. 4:**
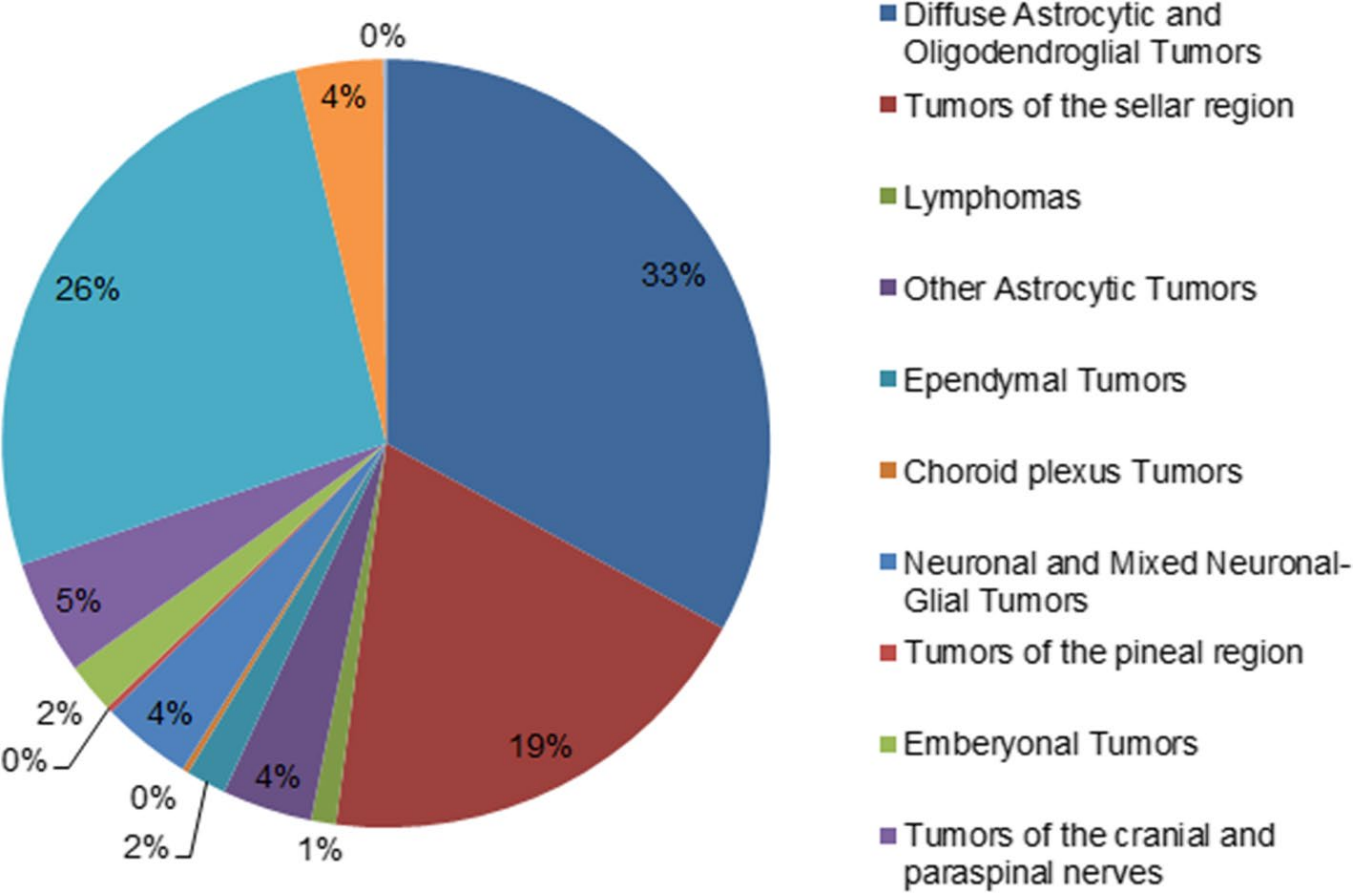
Distribution of the primary brain tumors by histopathology type

Diffuse astrocytic and oligodendroglial tumors were the most common histology group in our study with 252 cases (32.9%), followed by Meningiomas (26.4%), and tumors of the sellar region (19.1%). Glioblastoma was the most commonly identified histological type (18.8%), followed by pituitary adenoma (17.5%), and meningioma (16.5%). Among the malignant tumors, glioblastoma was the most common histologic type (48.8%). Meningiomas accounted for 42.8% of the total non-malignant tumors in our study, and pituitary adenoma accounted for 28.6% of them. Glioblastoma was more common in males (0.83 vs. 0.64, p-value < 0.001), while meningioma and pituitary adenoma were more common in females (0.35 vs. 0.94, and 0.65 vs. 0.73, respectively, *p*-value < 0.001). The distribution of PBTs by histopathology is presented in Table 6; Fig. 4.

**Table 6:** Distribution of the primary brain tumors by histopathology

Type	Code	Female (%)	IR	Male (%)	IR	Total (%)	IR
Diffuse Astrocytic and Oligodendroglial Tumors		103 (41)	1.07	149 (59)	1.52	252 (32.9)	1.30
Diffuse astrocytoma	9400/3	9 (100)	0.09	0 (0)	0	9 (1.2)	0.04
Gemistocitic astrocytoma	9411/3	3 (27.3)	0.03	8 (72.7)	0.08	11 (1.4)	0.06
Anaplastic astrocytoma	9401/3	4 (25)	0.04	12 (75)	0.12	16 (2.1)	0.08
Glioblastoma	9440/3	62 (43.1)	0.64	82 (56.9)	0.83	144 (18.8)	0.74
Giant cell glioblastoma	9441/3	0 (0)	0	1 (100)	0.01	1 (0.1)	0.005
Gliosarcoma	9442/3	1 (33.3)	0.01	2 (66.7)	0.02	3 (0.4)	0.02
Epitheliod glioblastoma	9442/3	1 (100)	0.01	0 (0)	0	1 (0.1)	0.005
Diffuse midline glioma	9384/3	0 (0)	0	2 (100)	0.02	2 (0.3)	0.01
Oligodendrogliomas	9450/3	7 (19)	0.07	30 (81)	0.31	37 (4.8)	0.19
Anaplastic oligodendroglioma	9451/3	8 (42.1)	0.09	11 (57.9)	0.11	19 (2.5)	0.10
Diffuse low-grade glioma	9452/1	4 (100)	0.04	0 (0)	0	4 (0.5)	0.02
Oligoastrocytoma	9382/3	3 (100)	0.03	0 (0)	0	3 (0.4)	0.02
Anaplastic oligoastrocytoma	9382/3	1 (50)	0.01	1 (50)	0.01	2 (0.3)	0.01
**Other Astrocytic Tumors**		**16 (55.2)**	**0.18**	**13 (44.8)**	**0.13**	**29 (3.8)**	**0.16**
Pilocytic astrocytoma	9421/1	15 (57.7)	0.16	11 (42.3)	0.11	26 (3.4)	0.14
Pleomorphic xanthoastrocytoma	9424/3	1 (33.3)	0.01	2 (66.7)	0.02	3 (0.4)	0.02
**Ependymal Tumors**		**5 (38.5)**	**0.05**	**8 (61.5)**	**0.08**	**13 (1.6)**	**0.07**
Subependymoma	9394/1	0 (0)	0	1 (100)	0.01	1 (0.1)	0.005
Ependymoma	9391/3	1 (33.3)	0.01	2 (66.7)	0.02	3 (0.4)	0.02
Supratentorial ependymoma	9391/3	0 (0)	0	1 (100)	0.01	1 (0.1)	0.005
Clear cell ependymoma	9393/3	1 (100)	0.01	0 (0)	0	1 (0.1)	0.005
Anaplastic ependymoma	9392/3	3 (42.9)	0.03	4 (57.1)	0.04	7 (0.9)	0.04
**Choroid plexus Tumors**		**1(50%)**	**0.01**	**1(50%)**	**0.01**	**2 (0.3)**	**0.01**
Choroid plexus papilloma	9390/0	1(50%)	0.01	1(50%)	0.01	2 (0.3)	0.01
**Neuronal and Mixed Neuronal-Glial Tumors**		**18 (62.1)**	**0.19**	**11 (37.9)**	**0.11**	**29 (3.7)**	**0.15**
Dysembryoplastic neuroepithelial tumor	9413/0	3 (75)	0.03	1 (25)	0.01	4 (0.5)	0.02
Ganglioglioma	9501/1	6 (75)	0.06	2 (25)	0.02	8 (1.0)	0.04
Papillary glioneuronal tumor	9505/1	3 (100)	0.03	0 (0)	0	3 (0.4)	0.01
Central neurocytoma	9506/1	5 (41.7)	0.05	7 (58.3)	0.07	12 (1.6)	0.06
Cerebellar liponeurocytoma	9506/1	1 (100)	0.01	0 (0)	0	1 (0.1)	0.005
Paraganglioma	8693/1	0 (0)	0	1 (100)	0.01	1 (0.1)	0.005
**Tumors of the pineal region**		2 (100)	0.02	0 (0)	0	**2 (0.2)**	0.01
Pineal parenchymal tumor of intermediate differentiation	9364/3	1 (100)	0.01	0 (0)	0	1 (0.1)	0.005
Pineoblastoma	9474/3	1 (100)	0.01	0 (0)	0	1 (0.1)	0.01
**Embryonal Tumors**		**10**	**0.10**	**6**	**0.06**	**16 (2.1)**	**0.082**
Medulloblastoma, classic	9470/3	6 (60)	0.06	4 (40)	0.04	10 (1.3)	0.05
Medulloblastoma, desmoplastic/nodular	9471/3	2 (66.7)	0.02	1 (33.3)	0.01	3 (0.4)	0.02
Medulloblastoma, large cell/ anaplastic	9473/3	2 (100)	0.02	0 (0)	0	2 (0.3)	0.01
Medulloblastoma, NOS	9470/3	0 (0)	0	1 (100)	0.01	1 (0.1)	0.005
**Tumors of the cranial and paraspinal nerves**		21 (56.8)	0.22	16(43.2)	0.16	**37 (4.8)**	0.19
Schwannoma	9560/0	21 (56.8)	0.22	16(43.2)	0.16	37 (4.8)	0.19
**Meningiomas**		**134(66.7)**	**1.38**	**67(33.3)**	**0.67**	**201 (26.4)**	**1.02**
Meningioma	9530/0	91 (72.2)	0.94	35 (27.8)	0.35	126 (16.5)	0.64
Meningiothelial meningioma	9530/1	5 (100)	0.05	0 (0)	0	5 (0.7)	0.02
Fibrous meningioma	9535/1	3 (60)	0.03	2 (40)	0.02	5 (0.7)	0.03
Transitional meningioma	9538/1	2 (28.6)	0.02	5 (71.4)	0.05	7 (0.9)	0.04
Psammomatous meningioma	9530/1	7 (87.5)	0.07	1 (12.5)	0.01	8 (1.0)	0.04
Angiomatous meningioma	9537/1	2 (66.7)	0.02	1 (33.3)	0.01	3 (0.4)	0.02
Microcystic meningioma	9532/1	3 (100)	0.03	0 (0)	0	3 (0.4)	0.02
Secretory meningioma	9530/0	3 (60)	0.03	2 (40)	0.02	5 (0.7)	0.025
Lymphoplasmacyte-rich meningioma	9537/1	3 (100)	0.03	0 (0)	0	3 (0.4)	0.02
Atypical meningioma	9539/1	14 (45.2)	0.15	17 (54.8)	0.17	31 (4.1)	0.16
Rhabdoid meningioma	9538/3	0 (0)	0	1 (100)	0.01	1 (0.1)	0.006
Anaplastic (malignant) meningioma	9531/3	1 (25)	0.01	3(75)	0.03	4 (0.5)	0.02
**Mesenchymal, non-meningiothelial Tumors**		**14 (50)**	**0.15**	**14 (50)**	**0.14**	**28 (3.6)**	**0.146**
Solitary fibrous tumor	8815/0	2 (40)	0.02	3 (60)	0.03	5 (0.6)	0.027
Heamangioblastoma	9161/1	2 (40)	0.02	3 (60)	0.03	5 (0.7)	0.025
Heamangioma	9120/0	8 (50)	0.09	8 (50)	0.08	16 (2.1)	0.084
Chondroma	9220/0	1 (100)	0.01	0 (0)	0	1 (0.1)	0.005
Angiosarcoma	9120/3	1 (100)	0.01	0 (0)	0	1 (0.1)	0.005
**Lymphomas**		**2 (25)**	**0.02**	**6 (75)**	**0.06**	**8 (1)**	**0.04**
Diffuse large B-cell lymphoma	9678/3	2 (28.6)	0.02	5 (71.4)	0.05	7 (0.9)	0.035
T-cell/histiocyte-rich large B-cell lymphoma	9688/3	0 (0)	0	1 (100)	0.01	1 (0.1)	0.005
**Germ cell Tumors**		**1 (100)**	**0.01**	**0 (0)**	**0**	**1 (0.1)**	**0.005**
Germinoma	9064/3	1 (100)	0.01	0 (0)	0	1 (0.1)	0.005
**Tumors of the sellar region**		**78 (53.4)**	**0.81**	**68 (46.6)**	**0.69**	**146 (19.1)**	**0.75**
Pituitary adenoma	8040/0	70 (52.2)	0.73	64 (47.8)	0.65	134 (17.5)	0.69
Craniopharyngioma	9350/1	6 (75)	0.06	2 (25)	0.02	8 (1.0)	0.04
Adamantinomatous craniopharyngioma	9352/1	2 (100)	0.02	0 (0)	0	2 (0.3)	0.01
Pituicytoma	9592/1	0 (0)	0	2 (100)	0.02	2 (0.3)	0.01

### First signs or symptoms for patients with PBTs

The most common first sign or symptom of PBTs among Palestinian patients was headache (41.4%), followed by focal signs (motor or sensory signs) (32.2%) and seizures (11%). The least common first signs or symptoms (0.1–0.7%) were dysarthria, neurogenic bladder/bowel, aphasia, and dysphagia. Among patients presenting first with focal signs, about 63.82% of them had a benign tumor, and 26.02% of them had a malignant tumor. Table 7 presents the first signs or symptoms for patients with PBTs.

**Table 7:** First sign or symptom by the behavior of the tumor

First sign/symptom	Total (%)	Benign	Unspecified, borderline, or uncertain behavior	Malignant	*p*-value
Headache	316 (41.4%)	140 (44.30%)	41 (12.97%)	135 (42.72%)	< 0.001
Seizure	85 (11%)	22 (25.88%)	15 (17.65%)	48 (56.47%)	
Focal signs (motor or sensory signs)	246 (32.2%)	157 (63.82%)	25 (10.16%)	64 (26.02%)	
Mental status alteration (drowsiness, confusion, etc.)	31 (4.1%)	8 (25.81%)	7 (22.58%)	16 (51.61%)	
Cognitive and emotional dysfunction	13 (1.7%)	3 (23.08%)	1 (7.69%)	9 (69.23%)	
Nausea/vomiting/dizziness	42 (5.5%)	19 (45.24%)	8 (19.05%)	15 (35.71%)	
Dysphagia	1 (0.1%)	0 (0%)	1 (100%)	0 (0%)	
Dysarthria	5 (0.7%)	2 (40%)	0 (0%)	3 (60%)	
Aphasia	2 (0.3%)	0 (0%)	0 (0%)	2 (100%)	
Neurogenic bladder/bowel	3 (0.4%)	1 (33.33%)	0 (0%)	2 (66.67%)	
Sexual dysfunction	20 (2.6%)	19 (95%)	0 (0%)	1 (5%)	

### Mortality rate

The total mortality rate from PBTs was 4.8%. Mortality was significantly associated with the age groups only. The mortality rate increases gradually from the 15–25 age group and reaches the highest level in the 76–85 age group. Despite their relatively rare occurrence, epitheliod glioblastoma, clear cell ependymoma, and solitary fibrous tumor were associated with 100% mortality rate. Medulloblastoma (large cell/ anaplastic) displayed the second highest mortality rate (50%), while both oligoastrocytoma and angiomatous meningioma had a mortality rate of 33% (p-value = 0.001). The mortality rate from primary brain tumors and its relation to sex, behavior of the tumor, histopathological types, and age groups is presented in Table 8.

**Table 8:** Mortality rate by other variables

Variable	Total	Deceased	Alive	*p*-value*
Sex:				
Male	359	12 (3.34%)	347 (96.66%)	0.069
Female	405	25 (6.17%)	380 (93.83%)	
**The behavior of the tumor**:				
Benign	371	19 (5.12%)	352 (94.88%)	0.679
Unspecified, borderline, or uncertain Behavior	295	3 (1.01%)	280 (98.99%)	
Malignant	98	15 (15.3%)	95 (84.7%)	
**Age Group**:				
15–25	96	2 (2.1%)	94 (97.9%)	0.004
26–35	140	2 (1.4%)	138 (98.6%)	
36–45	159	4 (2.5%)	155 (97.5%)	
46–55	158	8 (5.1%)	150 (94.9%)	
56–65	146	14 (9.6%)	132 (90.4%)	
66–75	57	6 (10.5%)	51 (89.5%)	
76–85	8	1 (12.5%)	7 (87.5%)	
**Histopathological type**:				
Meningioma	126	8 (6.3%)	118 (93.7%)	0.001
Glioblastoma	144	6 (4.2%)	138 (95.8%)	
Pituitary adenoma	134	7 (5.2%)	127 (94.8%)	
Pilocytic astrocytoma	26	2 (7.7%)	24 (92.3%)	
Anaplastic astrocytoma	16	2 (12.5%)	14 (87.5%)	
Oligoastrocytoma	3	1 (33%)	2 (67%)	
Atypical meningioma	31	1 (3.2%)	30 (96.8%)	
Cellular schwannoma	37	1 (2.7%)	36 (97.3%)	
Diffuse large B-cell lymphoma	7	1 (14.3%)	6 (85.7%)	
Transition meningioma	7	1 (14.3%)	6 (85.7%)	
Gemistocitic astrocytoma	11	1 (9%)	10 (91%)	
Anaplastic oligodendroglioma	19	1 (5.3%)	18 (94.7%)	
Angiomatous meningioma	3	1 (33%)	2 (67%)	
Medulloblastoma, large cell/ anaplastic	2	1 (50%)	1 (50%)	
Clear cell ependymoma	1	1 (100%)	0 (0%)	
Solitary fibrous tumor	1	1 (100%)	0 (0%)	
Epitheliod glioblastoma	1	1 (100%)	0 (0%)	
**Total**	764	37	727	4.8%

## Discussion

In many countries around the world, the incidence of PBTs and their subtypes is increasing and this could be explained by the raise in life expectancy of the population, increased access to healthcare, and availability of diagnostic imaging [13].

The mean age at diagnosis for the patients included in our study was 44.58 years (SD ± 15.27), which was lower than the mean age at diagnosis reported in previous studies in the USA [14], UK [15], France [16], and Lebanon [17], and higher than the mean age at diagnosis reported in a recent study in Jordan [6]. Among adolescents and adult Palestinians, the average annual incidence rate was 3.92 per 100,000. The incidence rate of PBTs in Palestine was lower than the worldwide overall incidence rate (10.82 per 100,000) [5]. When comparing the incidence rate in Palestine with the incidence rate in the Middle East, it was found to be lower than the incidence rate in Jordan (5.01 per 100,000) [6], and higher than the incidence rate in Qatar (2.2 per 100,000) and the UAE (0.56 per 100,000) [18, 19]. Moreover, the incidence rate of PBTs in Palestine was lower than the incidence rate in other countries like the USA (24.83 per 100,000) [14], Korea (23.39 per 100,000) [20], Austria (18.1 per 100,000) [21], Girona-Spain (16.85 per 100,000) [22], Georgia (10.62 per 100,000) [23], Lithuania (8 per 100,000), Norway (5.4 per 100,000), Australia (5.6 per 100,000), and Canada (5.3 per 100,000) [5]. On the other hand, the incidence rate of PBTs in Palestine was higher than the estimated incidence rate of brain tumors in Mexico (2.7 per 100,000) [24]. The gradual decrease in the incidence rate between 2020 and 2022 may be attributed to the COVID-19 pandemic, as during this period the transfer and admission rate for PBT decreased due to the movement restrictions and restricted entry to Jerusalem. The overall trend in the incidence of PBT needs further studies in the future to be determined.

The incidence rate in females was higher than in males. This result aligns with the findings of a previous study in Jordan [6]. The male-to-female ratio in our study was slightly lower than the ratio in Austria and Georgia [21, 23], and higher than Korea [20], UAE [19], and USA [14]. We found that most of the patients (79%) were older than 25 years and younger than 66 years. No significant differences in age groups between the males and females were found (p-value = 0.352). The distribution of the percentage of patients in the age groups was similar to the findings reported in a study in Northeast India and Qatar [18, 25].

The malignant PBTs in our study were fewer than the non-malignant tumors (38.61% vs. 61.39%). This finding aligns with the findings reported in previous studies in the USA [14], Georgia [23], UAE [19], and Korea [20]. Malignant tumors were more frequent than non-malignant tumors in Lebanon [17], England [15], and Spain [22]. The incidence rate of malignant tumors in Palestinian adolescents and adults was lower than the incidence rate of non-malignant tumors (1.52 vs. 2.41 per 100,000). This finding aligns with the findings reported in previous studies in the USA [14], and Georgia [23]. The incidence rate of malignant PBTs in Palestine was lower than the incidence rate of malignant tumors reported in the USA (6.94 per 100,000) [14], Korea (2.9 per 100,000) [20], and Iran (2.74 per 100,000) [26].

Meningiomas were the most common group of tumors identified in different studies around the world, including the USA (42.1%) [14], Korea (37.9%) [20], Lebanon (29.6%) [17], Northeast India [25], and Georgia [23], while in our study, the most common group of tumors identified in Palestinian adolescents and adults was diffuse astrocytic and oligodendroglial tumors (33%). In our study, the most commonly identified histopathological type was glioblastoma, while meningioma was the most common histopathological type identified in the USA [14], Korea [20], Jordan [6], Cameroon [27], and Austria [21].

Cerebral meninges were the most common location for PBTs identified in our study (26.3%), a finding that is similar to the findings of recent studies in Lebanon [17] and the USA [14]. Headaches, focal signs (motor or sensory signs), and seizures were the most common first signs or symptoms among Palestinian patients. These findings were similar to those reported in a recent study in Cameroon [27] and Saudi Arabia [28].

Unfortunately, no incidence rates of PBTs in Palestine during the years before 2018 were found in the literature, making the comparison and tracking the trend of annual incidence rates in Palestine impossible. We recommend conducting future studies that investigate the epidemiology of PBTs in Palestine including all the age groups of the population to provide constant information regarding the epidemiology of PBTs in Palestine. We also recommend using a unified methodology of similar epidemiological studies based on the WHO Classification of CNS tumors.

## Conclusion

This is the first national study that investigates the epidemiology of PBTs in Palestine. The study was conducted in Al-Makassed Hospital in Jerusalem, which serves as the largest referral center for primary brain tumors in Palestine. The overall incidence of PBTs in Palestinian adolescents and adults was 3.96 per 100,000, which was lower than the incidence rate of PBTs worldwide.

## Data Availability

The author confirms that all data generated or analysed during this study are included in this manuscript.
